# Gene therapy with tumor-specific promoter mediated suicide gene plus IL-12 gene enhanced tumor inhibition and prolonged host survival in a murine model of Lewis lung carcinoma

**DOI:** 10.1186/1479-5876-9-39

**Published:** 2011-04-11

**Authors:** Yu Xu, Jinxuan Hou, Zhengchun Liu, Haijun Yu, Wenjie Sun, Jie Xiong, Zhengkai Liao, Fuxiang Zhou, Conghua Xie, Yunfeng Zhou

**Affiliations:** 1 Department of Radiation and Medical Oncology, Zhongnan Hospital of Wuhan University, Wuhan 430071, PR China; 2 Hubei Key Laboratory of Tumor Biological Behaviors, Wuhan 430071, PR China; 3 Department of Oncology, Zhongnan Hospital of Wuhan University, Wuhan 430071, PR China

## Abstract

**Background:**

Gene therapy is a promising therapeutic approach for cancer. Targeted expression of desired therapeutic proteins within the tumor is the best approach to reduce toxicity and improve survival. This study is to establish a more effective and less toxic gene therapy of cancer.

**Methods:**

Combined gene therapy strategy with recombinant adenovirus expressing horseradish peroxidase (HRP) mediated by human telomerase reverse transcriptase (hTERT) promoter (AdhTERTHRP) and murine interleukin-12 (mIL-12) under the control of Cytomegalovirus (CMV) promoter (AdCMVmIL-12) was developed and evaluated against Lewis lung carcinoma (LLC) both *in vivo *and *in vitro*. The mechanism of action and systemic toxicities were also investigated.

**Results:**

The combination of AdhTERTHRP/indole-3-acetic acid (IAA) treatment and AdCMVmIL-12 resulted in significant tumor growth inhibition and survival improvement compared with AdhTERTHRP/IAA alone (tumor volume, 427.4 ± 48.7 mm^3 ^*vs *581.9 ± 46.9 mm^3^, *p *= 0.005 on day 15; median overall survival (OS), 51 d *vs *33 d) or AdCMVmIL-12 alone (tumor volume, 362.2 ± 33.8 mm^3 ^*vs *494.4 ± 70.2 mm^3^, *p *= 0.046 on day 12; median OS, 51 d *vs *36 d). The combination treatment stimulated more CD4^+ ^and CD8^+ ^T lymphocyte infiltration in tumors, compared with either AdCMVmIL-12 alone (1.3-fold increase for CD4^+ ^T cells and 1.2-fold increase for CD8^+ ^T cells, *P *< 0.01) or AdhTERTHRP alone (2.1-fold increase for CD4^+ ^T cells and 2.2-fold increase for CD8^+ ^T cells, *P *< 0.01). The apoptotic cells in combination group were significantly increased in comparison with AdCMVmIL-12 alone group (2.8-fold increase, *P *< 0.01) or AdhTERTHRP alone group (1.6-fold increase, *P *< 0.01). No significant systematic toxicities were observed.

**Conclusions:**

Combination gene therapy with AdhTERTHRP/IAA and AdCMVmIL-12 could significantly inhibit tumor growth and improve host survival in LLC model, without significant systemic adverse effects.

## Background

Over the last years, gene therapy has emerged as a promising strategy for cancer treatment [1]. However, some limitations are associated with its clinical application, the reduced specificity to deliver functional therapeutic genes into tumor cells being the major one [2]. Therefore, research in gene therapy has been focused on the development of targeting strategies.

Tissue- or cell-specific promoters represent one of the main methods of gene targeting. The human telomerase reverse transcriptase (hTERT) promoter has been widely used in gene therapy for targeting cancer cells, which is highly active in human cancer cells but not in normal differentiated human cells [3-6].Therefore, it fulfilled the characteristic of tumor origins with marked heterogeneity. Meanwhile, it was demonstrated that hTERT promoter had high transcriptional activity in a variety of human cancer cell lines, but not in normal human cells in adenovirus mediated transgene experiments [7,8]. Furthermore, Gu et al[9] showed that hTERT promoter could efficiently use mouse transcription machinery despite the apparent distinct regulatory mechanisms, and that hTERT promoter was highly active in murine tumor cells, but quiescent in normal murine cells and tissues. These findings indicated that hTERT promoter should be useful for targeting the pharmaceutical effects of a therapeutic gene to cancer cells.

Gene directed enzyme/prodrug therapy (GDEPT) or suicide gene therapy using viral vectors is an attractive alternative approach to cancer therapy, with the potential to give therapeutic ratios superior to standard chemo- and radiotherapy [10]. The horseradish peroxidase (HRP)/indole-3-acetic acid (IAA) system is a novel GDEPT system, which has shown great efficacy in killing tumor cells. In this setting, a viral vector expressing a therapeutic enzyme (HRP) is delivered to the tumor cells. The nontoxic prodrug (IAA) is administered systemically by intravenous injection or locally by intraperitoneal or intratumoral injection to maximize its concentration within the tumor, and converted into cytotoxic metabolites by HRP. It was demonstrated that HRP/IAA system was more cytotoxic to tumors than the well-known HSV-tk/GCV system [11,12]. Furthermore, HRP is normally absent in mammalian cells and IAA is a poor substrate for mammalian peroxidases, thus systemic toxicity is avoided [13].

To optimize the therapeutic efficacy of suicide gene therapy, it is important to explore new strategies of combined therapy, which employ targeted suicide gene in combination with immunotherapy that cooperatively enhance the antitumor effects while mitigating side effects. Immunotherapy uses the transfer of genes of various cytokines and co-stimulatory molecule into tumor cells to stimulate an antitumoral immune response in experimental animals [14]. Interleukin-12 (IL-12) is a heterodimetic cytokine, composed of 35 KDa (p35) and 40 KDa (p40) subunits, which bind to receptors present on NK and T cells [15]. IL-12 plays multiple roles in the immune system, such as augmenting the proliferation and cytotoxic activity of T cells and NK cells and initiating Th1-type immune responses by activation of CD4^+ ^and CD8^+ ^cells [16].

In the present study, we investigated a combined target suicide gene therapy and immunomodulating gene therapy approach for Lewis lung carcinoma (LLC), based on the delivery of HRP/IAA and murine IL-12 by the same adenovirus vector. These studies were performed both *in vitro*, by measuring cell viability, and *in vivo*, by determining the tumor size and animal survival, assessing both tumoral histology and infiltration of T-lymphocytes, and evaluating toxic studies. The data showed that combination gene therapy increased the therapeutic efficiency in the murine LLC model used in this study.

## Materials and methods

### Cell culture and animals

LLC and A549 cell lines were obtained from the Cell Bank of the Chinese Academy of Science (Shanghai, China) and maintained in 5% CO_2 _at 37°C in Dulbecco's minimum essential medium (DMEM) containing 10% fetal bovine serum (FBS), 100 U/ml penicillin and 100 mg/ml streptomycin. All culture reagents were purchased from Hyclone (Logan, UT, USA) or Invitrogen (Gaithersburg, MD, USA). Being syngenic with LLC, male C57BL/6 mice (6-week old) obtained from Shanghai SLAC Laboratory Animal Co. Ltd (Shanghai, China) were housed in specific pathogen-free condition at the Animal Experimental Center of Wuhan University. The facilities and the protocol were consistent with the regulations on animal use for biomedical experiments issued by the Ministry of Science and Technology of China, and approved by the Animal Care Committee of Wuhan University.

### Recombinant adenoviruses

The plasmid phTERTHRP constructed in our lab as described [17-19] was digested with MluI and BamHI, and subcloned into the same site of pAdTrack-C (which was modified by inserting MluI and BamHI clone sites based on pAdTrack) to generate pAdhTERTHRP. The murine IL-12 obtained from pUMVC3-mIL12 (Aldevron Inc., Fargo, USA) was subcloned into pAdTrackCMV to generate pAdCMVmIL-12 by digesting with SalI and NotI. The shuttle vector pAdTrack and *Escherichia coli *AdEasy-1 were kindly provided by Dr. JG Wu (State Key Laboratory of Virology, College of Life Sciences at Wuhan University, China). For recombinant prAdhTERTHRP and prAdCMVIL-12, homologous recombination was performed as described previously [20]. Recombinant adenoviruses were packaged by GeneChem Co., Ltd (Shanghai, China). Briefly, recombinant plasmids were transfected into 293 cells to obtain adenovirus prestocks. Virus was purified by double cesium chloride gradient ultracentrifugation. Viral titer was determined by plaque assay and expressed as plaque-forming units (pfu). Purified virus aliquots were stored at -80°C.

### *In vitro *studies

For adenoviral gene transduction efficiency *in vitro*, A549 and LLC cells were infected with AdCMV(-) at multiplicity of infection (MOI) of 1, 10, 100 and 1,000. After incubation for 48 hours, the cells were analyzed using flow cytometry (FC500, Beckman coulter, CA, USA) for green fluorescent protein (GFP) expression. Subsequently, LLC cells were transduced with AdCMV(-), AdCMVmIL-12 and AdhTERTHRP alone or in combination at appropriate MOI. Cell proteins were harvested and the expression of HRP was detected by western blot. Culture supernatants were collected for determination of IL-12 concentration by a sandwich enzyme-linked immunosorbant assay (ELISA).

For cytotoxicity of HRP/IAA system, LLC cells (2 × 10^3^/well) were plated in 96-well plates and allowed to adhere overnight. The cells were transduced as described above and incubated for 16 hours. Then fresh media containing IAA (Sigma, MO, USA) at a concentration of 0-5 mM were exchanged every 48 hours. Cell viability was determined by MTT assay (Invitrogen, CA, USA) 120 hours later and the optical density value was measured by a microplate reader (Turner BioSystems, CA, USA).

### *In vivo *studies

**Figure 1:**
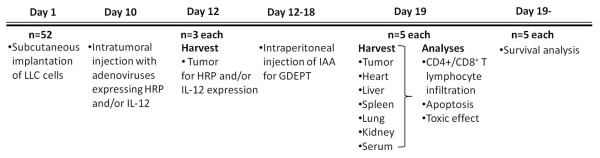
**General outline of the *in vivo *experimentation with mouse model of LLC**.

A total of 5×10^6 ^LLC cells were inoculated subcutaneously in the right flank of C57BL/6 mice. After 14 days, the tumor was isolated, prepared to cell suspension and inoculated into new mice. When the tumors reached 5-6 mm in diameter (Day 10), the mice were randomized to 4 groups (n = 13 each): group I, AdCMV(-) (1 × 10^9 ^pfu); group II, single-agent AdhTERTHRP (5 × 10^8 ^pfu); group III, single-agent AdCMVmIL-12 (5 × 10^8 ^pfu); group IV, combination AdCMVmIL-12 and AdhTERTHRP (5 × 10^8 ^pfu + 5 × 10^8 ^pfu). The adenoviruses were diluted in 30 μl phosphate buffered saline (PBS). 48 hours after virus injection (Day 12), 3 mice of each group were sacrificed for examining HRP and IL-12 expression in tumor tissues. Meanwhile, IAA (50 mg/kg daily) was administered to the rest mice by intraperitoneal injection for 7 days from Day 12 to 18. Five mice from each group were sacrificed for evaluating the effects of various treatments on Day 19. In addition, survival studies were set up with different treatment groups of animals (n = 5) in an identical manner. Tumor size was measured using caliper every 3 days and the volume was calculated using the following formula: (L × W^2^)/2, where L equals length and W equals width. Animals with very high tumor volume (exceeded 3500 mm^3^) were sacrificed for ethical reasons and this was recorded as the date of death for survival studies. The general scheme of *in vivo *experiment was outlined in Figure 1.

### Western blot analysis

HRP expression in LLC cells and subcutaneous tumors infected with AdCMV(-), AdhTERTHRP and AdCMVmIL-12 alone or in combination was determined by Western blot. Transduced cells and tumor tissues were lysed in 2× sample buffer (100 mM Tris-HCl pH6.8, 200 mM DTT, 4% SDS, 20% glycerol and 0.2% bromoplenol blue) and separated by 10% SDS-PAGE. Proteins were transferred to PVDF membranes (Millipore, MA, USA) and then immersed in a blocking solution containing 5% non-fat milk and 0.1% tween-20 for 1 hour. Afterwards, the membranes were incubated with mouse anti-HRP (dilution, 1:500) or mouse anti-β-actin (dilution, 1:1000) for 2 hours and with goat anti-mouse secondary antibody (dilution, 1:10000) for 1 hour at room temperature. All the antibodies were purchased from Santa Cruz Biotechnology (Santa Cruz, USA). Enhanced chemiluminescence (Beyotime, Shanghai, China) was used to visualize the immunoreactive bands.

### ELISA

Culture supernatants of transduced LLC cells and tumor tissue lysates of treated mice were collected. The IL-12 concentration was determined using a sandwich ELISA (R&D systems, CA, USA) according to the manufacturer's instructions.

### Immunohistochemical analysis and apoptosis assay

Tumor tissues were formalin fixed and 4 μm sections were stained with hematoxylin and eosin for routine histological analysis. For immunohistochemical analysis, acetone fixed fresh-frozen sections were stained for infiltration T lymphocytes (CD4^+ ^and CD8^+^) with specific antibodies (BD PharMingen, CA, USA) following standard method as described [21]. For apoptosis assay, formalin fixed sections were analyzed for DNA fragmentation by terminal deoxynecleotidyl transferase-mediated dUTP nick-end labeling (TUNEL) assay (Roch, NJ, USA) according to the manufacturer's instructions. Cell proliferation was also determined using anti-Ki-67 antibody (Santa Cruz Biotechnology, Santa Cruz, USA). Positive staining was scored by light microscopy. After initial scanning under × 100 magnification, positively stained cells in ten fields under × 400 (0.15 mm^2^) magnification were counted and the mean number/high power field (HPF ± SEM) was determined.

### *In vivo *toxicity studies

Sera were collected from the treated animals to measure the biochemistry markers including alanine transaminase (ALT), aspartate aminotransferase (AST), blood urea nitrogen (BUN) and creatine (Cr) using commercial kits (Sigma, MO, USA). For histological examination, some tissues were harvested, fixed with formalin and stained with hematoxylin and eosin. 3 mice without any treatment were used as normal control.

### Statistical analysis

The significance of differences between experimental groups was calculated using Student's t-test or one-way ANOVA analysis as appropriate. Kaplan-Meier curves were compared using the log-rank test. In all cases, *P *values less than 0.05 were considered statistically significant. Analysis was performed with the GraphPad Prism 5 (version 5.01, GraphPad software, Inc.).

## Results

### Gene transfer efficiency in LLC cells with Adenoviruses

**Figure 2:**
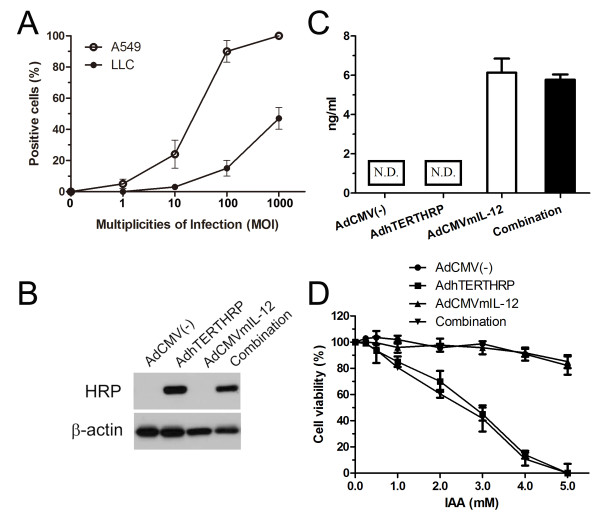
**Transduction of adenoviruses in LLC cells *in vitro***. A, adenoviral gene transduction efficiencies in murine LLC and human A549 cell lines at indicated MOIs. B, HRP expression of LLC cells in various treatment groups was analyzed by western blot. C, IL-12 expression in the supernatant of LLC cells infected with AdCMV(-), AdhTERTHRP and/or AdCMVmIL-12 was quantified using mIL-12p70 ELISA kit following the manufacturer's instructions. N.D., no detection. D, effects of transduction with different adenoviruses followed by IAA treatment in LLC cells *in vitro*. Transduced cells were incubated with various concentrations of IAA for 5 days, and cell viability was determined by MTT assay. IC_50 _was calculated as the concentration of drug which inhibited cell growth by 50%. Data were representative of three independent experiments. Each point represented the means ± SEM and was expressed as percentage relative to drug-free cells.

LLC cells were relatively resistant to infection by AdCMV(-) compared with A549 cells. Only a few LLC cells were infected at MOI of 10. When the MOI increased to 100 and 1000, the percentages of transduced cells were up to 10-15% and 40-45% (Figure 2A). Thus the MOI of 1000 was chosen for the subsequent studies.

### HRP and IL-12 expression *in vitro*

LLC cells transduced with AdCMV(-), AdhTERTHRP and AdCMVmIL-12 alone or in combination were harvested and determined by western blot for the expression of HRP. Results showed that the HRP expression was observed in AdhTERTHRP alone group and combination group whereas not in AdCMV(-) or AdCMVmIL-12 alone groups (Figure 2B). The expression levels of IL-12 in culture supernatant were measured by ELISA assay. LLC cells in AdCMVmIL-12 alone and in combination groups resulted in secretion of up to 5.8 ng/ml, but no IL-12 expression was detected in supernatants from AdCMV(-) or AdhTERTHRP alone groups (Figure 2C).

### Cytotoxicity of the HRP/IAA system *in vitro*

To validate the biological activity of exogenous HRP, transduced LLC cells were treated with IAA at indicated concentrations in Figure 2D. The results were shown as the percentage of cell viability with respect to control cells without IAA treatment. LLC cells in AdhTERTHRP alone group and combination group exhibited a dose-dependent manner of cytotoxicity, and both the IC_50 _of IAA was about 3.0 mM. The results indicated that HRP/IAA system had efficient cytotoxic effects on LLC cells. However, high concentration of IAA (more than 5 mM) showed mild toxicity on HRP-negative cells.

### *In vivo *antitumor effect of gene therapy

**Figure 3:**
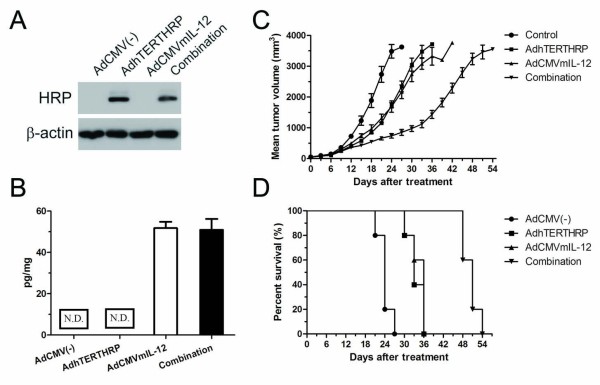
***In vivo *evaluation of antitumor effect in a murine model of LLC**. A, HRP expression in tumor tissues of various treatment groups was analyzed by western blot. B, IL-12 expression in tumor tissue lysates of different treatment groups was quantified by ELISA. N.D., no detection. C, tumor volumes were measured at the indicated time points after intratumoral injection with AdCMV(-), AdhTERTHRP, AdCMVmIL-12 alone or in combination. Each data point represented the mean tumor volume in that group. Error bars represented means ± SEM. B, long-term survival of animals after treatment with different strategies. (n = 5 per group).

The LLC mouse model was used to assess the *in vivo *antitumor activity of AdhTERTHRP and AdCMVmIL-12 as single agent or in combination. First of all, the expression of HRP and IL-12 in tumors was determined and similar results were found as *in vitro *(Figure 3A and 3B). For tumor growth, the data clearly showed remarkable inhibition of combining AdCMVmIL-12 and AdhTERTHRP treatment in comparison with AdCMV(-), AdCMVmIL-12 and AdhTERTHRP alone (Figure 3C). AdhTERTHRP treatment (group II) significantly suppressed tumor growth through day 6 to 24 compared with AdCMV(-) treatment (group I) (groups II *vs *I, *p *= 0.024). AdCMVmIL-12 treatment (group III) was associated with more potent antitumor effects (groups III *vs *II, *p *> 0.05; groups III *vs *I, *p *= 0.047 on day 9 to 24). Combination treatment with AdhTERTHRP and AdCMVmIL-12 (group IV) was associated with the most marked suppression of tumor growth (groups IV *vs *III, *p *= 0.046 on day 12 to 24; groups IV *vs *II, *p *= 0.005 on day 15 to 24; groups IV *vs *I, *p *= 0.029 on day 6 to 24). These results indicated that both AdhTERTHRP alone and AdCMVmIL-12 alone suppressed tumor growth, and the combination showed synergistic antitumor effects. As a consequence, five animals from each group were monitored and survival curves were established (Figure 3D). Mice treated with the combination regimen had a significant survival advantage with the median survival increase to 51 versus 36 days for AdCMVmIL-12 alone-treated mice, 33 days for AdhTERTHRP alone-treated mice and 24 days for AdCMV(-) control treated mice (Figure 3B). Statistical comparison (log-rank test) showed a significant difference (*P *= 0.0001) and a significant trend between treatment groups (*P *= 0.0003).

### T lymphocyte infiltration in tumors

**Figure 4:**
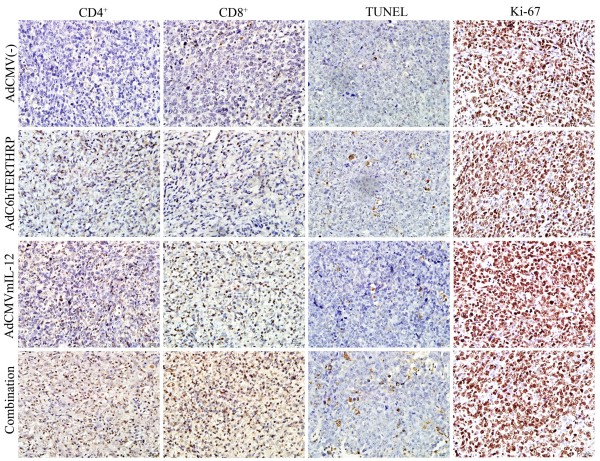
**Effects of different treatments on tumor infiltration by immune cells, apoptosis and proliferation in LLC tumors**. Treated mice were sacrificed 24 hours after last IAA administration. Tumor sections were determined by immunohistochemical staining using specific antibodies against CD4, CD8, Ki-67 and TUNEL assay. The positive cells were scored through light microscopy. After initial scanning under × 100 magnification, positive stained cells in 10 filed under × 400 magnification were counted and the mean number of stained cells was averaged over 10 fields.

The antitumoral activity of immuno-gene therapy strategies involved the activation of the immune system against the neoplastic tissue. To evaluate the effects of different treatments on immune cell infiltration in local tumors, immunohistochemistry was performed against CD4^+ ^and CD8^+ ^T cells (Figure 4; Table 1). The results revealed that the combination therapy (AdhTERTHRP + AdCMVmIL-12) led to extensive tumor infiltration by CD4^+ ^T cells (*P *< 0.0001, 9.4-fold) and CD8^+ ^T cells (*P *< 0.0001, 8.6-fold) compared with AdCMV(-) group. The infiltration of CD4^+ ^and CD8^+ ^T cells in combination group was also substantially greater than that observed in tumors given either AdCMVmIL-12 (*P *= 0.002, 1.3-fold for CD4^+ ^T cells; *P *= 0.001, 1.2-fold for CD8^+ ^T cells) or AdhTERTHRP (*P *< 0.0001, 2.1-fold for CD4^+ ^T cells; *P *< 0.0001, 2.2-fold for CD8^+ ^T cells) alone.

**Table 1:** Analyses of LLC tumor sections showing effects of different treatments on tumor infiltration by immune cells and apoptosis

Treatment group	Mean number/HPF ± SEM╪			
	
	Th cells (CD4)	Cytolytic T cells (CD8)	Apoptotic cells (TUNEL)	Proliferating cells (Ki-67)
AdCMV(-)	14.8 ± 2.8	20.6 ± 4.9	2.1 ± 1.2	437.4 ± 39.7
AdhTERTHRP	67.2 ± 7.7	79.1 ± 9.4	25.8 ± 3.3	471.5 ± 51.7
AdCMVmIL-12	109.6 ± 11.0	143.1 ± 38.7	14.4 ± 1.7	409.6 ± 36.1
Combination	139.1 ± 17.6	177.2 ± 12.6	40.2 ± 4.4	463.3 ± 55.4
P*	< 0.0001	< 0.0001	< 0.0001	> 0.05

╪After initial scanning under × 100 magnification, positive stained cells in 10 fields under × 400 (0.15 mm^2^) magnification were counted and the mean number/high-power field (HPF) was determined.

*Statistical analyses were conducted with one-way ANOVA for all the parameters. *P *values less than 0.05 were considered statistically significant.

### Apoptosis and proliferation in tumors

To examine potential mechanism of treatment-related antitumor effects, apoptosis and proliferation were assessed in tumors from different treatment groups. Apoptotic cells with brown nuclei were counted under a light microscope in randomly chosen fields. The results showed that a significant increase in the apoptotic cells in combination group (*P *< 0.0001, Figure 4; Table 1) compared with all other groups. The trend, combination > AdhTERTHRP > AdCMVmIL-12 > AdCMV(-), implied that the apoptosis inducing effects of combination strategy were more potent than those induced by either AdCMVmIL-12 (*P *< 0.001, 2.8-fold) or AdhTERTHRP (*P *< 0.001, 1.6-fold) alone in LLC tumors. However, analysis of cell proliferation using anti-Ki-67 antibody staining in tumors did not show any significant differences between groups.

### *In vivo *toxicity studies

**Figure 5:**
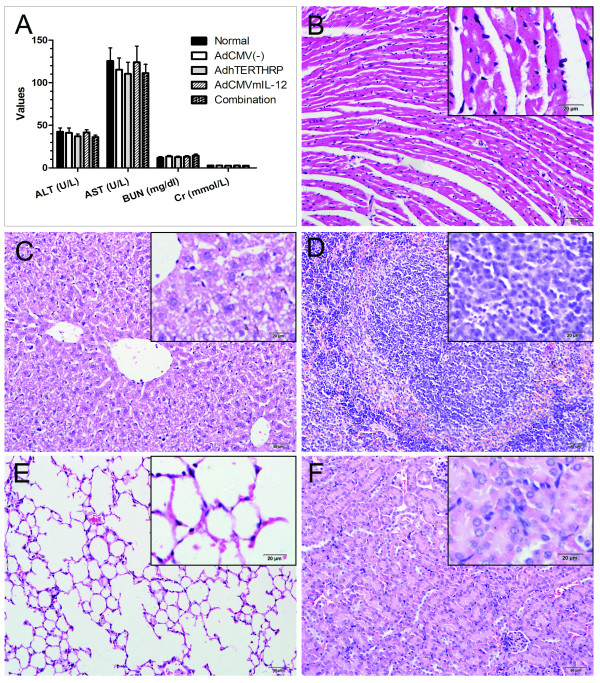
***In vivo *toxicity studies**. A, biochemistry markers in serum. The data were presented as means ± SEM of fiver animals per group. B-F, representative images for histology of treated heart (B), liver (C), spleen (D), lung (E) and kidney (F) from combination group. (B-F), Magnification, × 200; Scale bar, 50 μm. (B-F inserts), Magnification, × 400; Scale bar, 20 μm.

To evaluate *in vivo *toxicity of various strategies with adenoviruses, biochemistry markers of liver and kidney in sera and histological changes of key tissues were examined from treated mice. The results showed that the liver and kidney function was not impaired in each treatment group (Figure 5A). Meanwhile, there were no obvious pathological changes in heart, liver, spleen, lung and kidney of treated mice in comparison with animals without treatment (Figure 5B-F). Neither serum markers nor histology differed between virus treatment groups versus normal control mice, suggesting that intratumoral administration with AdhTERTHRP and/or AdCMVmIL-12 did not cause detectable system toxicity.

## Discussion

Gene therapy has been used extensively to cure a variety of tumors in different experimental models [22]. However, the specificity of therapeutic gene expression was unsatisfied [4]. To prevent the toxicity of suicide genes in normal cells, tumor specific promoters including hTERT promoter have been utilized to drive the specific expression of 'toxic' genes in tumors of certain origins [23-25]. hTERT is transcriptionally repressed in normal human adult tissues but up-regulated in the majority of human tumors from all tissues, which prompted the investigations on the use of hTERT promoter to restrict the expression of delivered genes to cancer cells and the results were encouraging [4,26-28].

Suicide gene delivered by viral vectors was demonstrated to be an effective approach for cancer treatment. Besides direct killing effect on transduced cells, the bystander effect of suicide gene therapy plays a crucial role in cancer treatment due to it is impossible to transfer the suicide gene into all tumor cells. Meanwhile, it was reported that the the host's immune system plays an important role in the bystander effect *in vivo *[29,30]. Thus, the improvement of host's immunity could enhance the bystander effect of the suicide gene therapy. IL-12 is an important macrophage-derived cytokine that can drive IFN-γ production, which exerted direct effects on the tumor or recruited endogenous APCs (antigen present cells) and effector T cells to the tumor site [31,32]. Local expression of IL-12 (to maintain low serum concentrations to reduce systemic toxicity) could be readily achieved by gene therapy vectors [33]. Local concentration of IL-12 could not only reduce toxicity but might be crucial for the establishment of antitumoral immunity [34].

Therefore, strategies combined suicide gene with immune-gene could enhance the antitumor effect than either alone, which was demonstrated in several experimental models [15,16,35,36]. In the present study, we evaluated the therapeutic efficiency of target suicide gene therapy mediated by hTERT promoter in combination with immuno-gene therapy in a murine model of LLC both *in vitro *and *in vivo*.

The suicide gene (AdhTERTHRP) and immune-gene (AdCMVmIL-12) were constructed based on adenovirus due to its high transgene efficiency. However, the results showed that murine LLC cells were relatively resistant to adenovirus infection compared with human A549 cells (Figure 2A), which was consistent with previous reports [37]. The present study employed LLC cell line since it is syngenic with the immunocompetent mouse model. The expression of HRP protein was detected in AdhTERTHRP alone and combination groups but not in AdCMV(-) or AdCMVmIL-12 alone groups *in vitro *(Figure 2B) and *in vivo *(Figure 3A). In the contrast, IL-12 was detected in culture supernatant (Figure 2C) and tumor tissues (Figure 3B) in AdCMVmIL-12 and combination groups while not in AdCMV(-) and AdhTERTHRP groups. The results indicated that therapeutic genes were successfully delivered by adenovirus vectors and the tumor specific promoter (hTERT) was efficient to drive target gene expression. In addition, the HRP/IAA system was reported to be more cytotoxic to tumor cells than the well-known HSV-tk/GCV system, especially in the anoxia condition [11,12]. Strong cytotoxicity of HRP/IAA system *in vitro *was also observed in the study with a dose dependent manner, which further suggested the biological activity of HRP coded by exogenous genes (Figure 2D).

For *in vivo *study, formation of tumor nodule and transgene expression before IAA administration in the study indicated that the present experimental system might mimic some clinical situations and might be very suitable for the purpose of assessing therapeutic effects. The combination of AdhTERTHRP with AdCMVmIL-12 not only showed significantly stronger tumor suppression effects (Figure 3C), but also remarkably prolonged the survival of animals (Figure 3D) compared with AdCMVmIL-12 alone, AdhTERTHRP alone and AdCMV(-).These findings taken together with the fact that the tumor specific hTERT promoter was sufficient to drive suicide gene expression indicated that adenovirus-mediated HRP gene therapy combined with cytokine IL-12 gene therapy might be clinically therapeutic and useful for lung cancer.

We further investigated the possible mechanisms of the enhanced antitumor effects by HRP/IAA and IL-12 combination gene therapy (Figure 4, Table 1). The results indicated that the activities of HRP/IAA could not only generate significantly cytotoxic activities locally, but also potentially maximize tumor antigen presentation through its necrotic and apoptotic effects. The addition of adenoviral vector-delivered locally active IL-12 could potentially maximize the infiltration of specific immune cells and cause them to be activated and mature into active effectors, and thus effectively co-operate with HRP/IAA gene therapy. Another major focus of the present study was to evaluate whether suicide gene therapy plus immuno-gene therapy had any toxicity on the treated animals. The results showed that there was no obviously systemic toxicity, as shown by serum analysis for biochemical markers of liver and kidney and the histological examination of hematoxylin and eosin stained major organs (Figure 5). Taking all these data into consideration, it appears that combination therapy of AdhTERTHRP/IAA and AdCMVmIL-12 had the best therapeutic effect in terms of tumor growth, survival in the LLC tumor model and without detectable system toxicity.

## Conclusions

In summary, the concept of using targeted suicide gene therapy in combination with immuno-gene therapy is attractive for many malignancies. The present study could be concluded that the combination therapy of HRP/IAA and IL-12 were able to reduce the tumor growth and enhanced animal survival in the LLC model. This combined system could provide a more effective and less toxic therapy for cancer, although further studies and clinical trials will be necessary in the future.

## List of abbreviations

hTERT: human telomerase reverse transcriptase; GDEPT: Gene directed enzyme/prodrug therapy; HRP: horseradish peroxidase; IAA: indole-3-acetic acid; IL-12: interleukin-12; CMV: Cytomegalovirus; LLC: Lewis lung carcinoma; DMEM: Dulbecco's minimum essential medium; FBS: fetal bovine serum; pfu: plaque-forming units; MOI: multiplicity of infection; ELISA: enzyme-linked immunosorbant assay; TUNEL: terminal deoxynecleotidyl transferase-mediated dUTP nick-end labeling; ALT: alanine transaminase; AST: aspartate aminotransferase; BUN: blood urea nitrogen; Cr: Creatinine.

## Conflict of interest

The authors declare that they have no competing interests.

## Authors' contributions

YX selects the research topic, conducts most experiments, statistical analysis and writes manuscript. JH and ZL conduct the pathological examination. HY, WS, JX, ZL, FZ and CX supply technique assistance and review the manuscript. YZ conceives the study project, organizes the whole study process, provides financial support, and finalizes the manuscript. All authors have read and approved the final manuscript.
